# Impact of phages on soil bacterial communities and nitrogen availability under different assembly scenarios

**DOI:** 10.1186/s40168-020-00822-z

**Published:** 2020-04-06

**Authors:** Lucas P. P. Braga, Aymé Spor, Witold Kot, Marie-Christine Breuil, Lars H. Hansen, João C. Setubal, Laurent Philippot

**Affiliations:** 1 Université Bourgogne Franche-Comté, INRA, AgroSup Dijon, Agroécologie, 21000 Dijon, France; 2 Departamento de Bioquímica, Instituto de Química, Universidade de São Paulo, São Paulo, Brazil; 3 Department of Plant and Environmental Sciences, University of Copenhagen, Frederiksberg, Denmark

## Abstract

**Background:**

Bacteriophages, the viruses infecting bacteria, are biological entities that can control their host populations. The ecological relevance of phages for microbial systems has been widely explored in aquatic environments, but the current understanding of the role of phages in terrestrial ecosystems remains limited. Here, our objective was to quantify the extent to which phages drive the assembly and functioning of soil bacterial communities. We performed a reciprocal transplant experiment using natural and sterilized soil incubated with different combinations of two soil microbial communities, challenged against native and non-native phage suspensions as well as against a cocktail of phage isolates. We tested three different community assembly scenarios by adding phages: (a) during soil colonization, (b) after colonization, and (c) in natural soil communities. One month after inoculation with phage suspensions, bacterial communities were assessed by 16S rRNA amplicon gene sequencing.

**Results:**

By comparing the treatments inoculated with active versus autoclaved phages, our results show that changes in phage pressure have the potential to impact soil bacterial community composition and diversity. We also found a positive effect of active phages on the soil ammonium concentration in a few treatments, which indicates that increased phage pressure may also be important for soil functions.

**Conclusions:**

Overall, the present work contributes to expand the current knowledge about soil phages and provide some empirical evidence supporting their relevance for soil bacterial community assembly and functioning.

Video Abstract

## Background

Bacteriophages (or simply phages), the viruses that infect bacteria, modulate ecological and evolutionary processes in microbial communities by complex antagonistic and mutualistic coevolutionary interactions [1–3]. Through antagonistic interactions, phages control host population size by lytic infections [4] and promote microbial biomass turnover over time by releasing nutrients trapped in microbial biomass (i.e., the viral shunt [5]). Many phages are also capable of lysogenic infection, which consists in the incorporation of phage DNA into the host genome. The presence of phage DNA can affect bacterial genomes in different ways, which may lead to changes in the host phenotype [6, 7]. Such genomic interactions can shape the evolution of microbial metabolic pathways and also affect biogeochemical cycles [8].

The ecological relevance of phages for microbial systems has mostly been studied in marine environments. It was estimated that approximately 20% of the oceanic microbial biomass are killed by phages daily and that around 3 Gt of carbon per year is released through the viral shunt [9, 10]. Accordingly, the relative abundance of viral genes is acknowledged as the best predictor of the global carbon flux in the deep sea [11]. The positive impact of host cell lysis by phages on nutrient dynamics in marine environments is well recognized [12, 13]. On the other hand, it is likely that bacterial mortality due to phage lysis can also slow nutrient transformations if key bacteria species that normally mediate these transformations experience significant population reduction. Hundreds of thousands of new viral populations have been uncovered with the analysis of marine viromes [8, 14, 15]. Evidences about the crucial role of phages on ecosystems functions start also to accumulate for other environments such as fracking systems [16], rumen [17, 18], human gut [19], mangrove [20], and wetlands [21].

In soil, metagenomic analysis of permafrost ecosystems has recently demonstrated that phages can infect key carbon cycling microbes and may impact on biogeochemical cycles [22, 23]. Estimates based on direct counts indicate that soils can contain a high number of phage (up to ~ 10^10^ per gram of soil [24]), whose abundance are affected by land use as well as soil moisture and temperature [25]. However, despite the accumulated evidences in the other systems, the importance of phages for bacterial communities in terrestrial ecosystems remains unknown. In a recent review, Kuzyakow and Mason-Jones [26] suggested that the rate of infection by phage may even be higher in soil that in aquatic ecosystems because of more frequent physical encounters between phages and bacteria in soil.

In the present study, we performed soil manipulation experiments in order to assess the importance of the lytic effect of phages for the assembly of soil bacterial communities. For this purpose, we used a reciprocal soil phages transplant design under different community assembly scenarios. Considering that coevolutionary interactions between phages and bacteria are stated in context of local adaptation [2], we first hypothesized that addition of phages will lead to stronger shifts in microbial community composition for non-native communities (from a different soil) than native communities (from the same soil). Given that already-established bacteria can develop localized microcolonies, better occupy the soil aggregates, and likely be more protected from predation [27, 28], we also hypothesized that phage addition has a stronger effect on community composition when bacteria are colonizing the soil than when bacterial communities are already established in the soil. Finally, we assessed whether soil functioning was affected by phage addition using N-cycling as a model function. We focused on nitrogen cycling because nitrogen is the major nutrient limiting primary production in terrestrial ecosystems [29]. Among Earth-system processes, the nitrogen cycle is also one which was pushed by human activities outside critical thresholds representing the safe operating space [30].

## Results

### Differences in bacterial communities between natural soils

To test the effect of phages on their native and non-native soil bacterial communities, we used two different soils (S1 and S2), sampled from agricultural systems, to setup microcosms in a reciprocal transplant design under different community assembly scenarios. Our results indicate that bacterial community diversity was significantly different between soils S1 and S2 (Additional file 1: Fig. S1). Alpha diversity analysis revealed that taxon richness was higher in S1 than S2 (Additional file 1: Fig. S1a; *p* value = 0.016) and that taxa were more evenly distributed in S2 than S1 (Additional file 1: Fig. S1b; *p* value = 0.009). These differences between bacterial communities were also confirmed by beta diversity analysis (Additional file 1: Fig. S1d, *p* value = 0.011, and Additional file 1: Fig. S1e, *p* value = 0.016).

### Characterization of phages in the S1 and S2 soil suspensions

S1 and S2 suspensions were filtered using a tangential filter flow (TFF) system to obtain the phage suspensions (PS1 and PS2) for further manipulations (see “Materials and methods”). The phage suspensions were investigated using transmission electron microscopy (TEM) and metagenomics. The microscopy images obtained from PS1 and PS2 confirmed the presence and the integrity of phages obtained in the soil suspensions (Additional file 2: Fig. S2ab). We observed the presence of tailed phages (*Caudovirales* order) (Additional file 2: Fig. S2ab). The observation of morphological features (i.e. capsid sizes) indicates an intra-clade diversity within *Caudovirales* in both soils.

Only a few bacterial cells were observed in TEM images. Consistent with that, only few contigs from bacterial genomes were recovered from the metagenomic datasets. Those contigs were assembled into three bins that were classified as *Stenotrophomonas maltophilia*, *Delftia acidovorans*, and an unknown member species of the Caulobacteraceae family. Together, their relative abundance represented only 1.18 % (± 1.14) across the datasets (i.e., more than 97.8 % of mapped reads were phage genomic sequences).

Phage genome recovery from the metagenomic datasets resulted in 151 bins representing distinct phage populations. S1 presented a higher overall abundance of reads mapped to phage genomic sequences compared to S2, with 72 bins being more abundant in the first and 39 in the latter (Additional file 2: Fig. S2c, *t* test *p* value ≤ 0.05). Ten of these bins were detected only in S1 (Additional file 2: Fig. S2c). The classification using vConTACT2 was able to cluster significantly (*p* value < 0.05) 14 of the phage bins with known phage genomes. The genomes clustered with the phage bins belong to the clades of Escherichia phage, Gordonia phage, Mannheimia phage, Mycobacterium phage, and Streptomyces phage. These results indicate that the majority of the phages obtained from the soil suspensions belong to undescribed phage clades.

### Impact of phages on bacterial communities

**Fig. 1:**
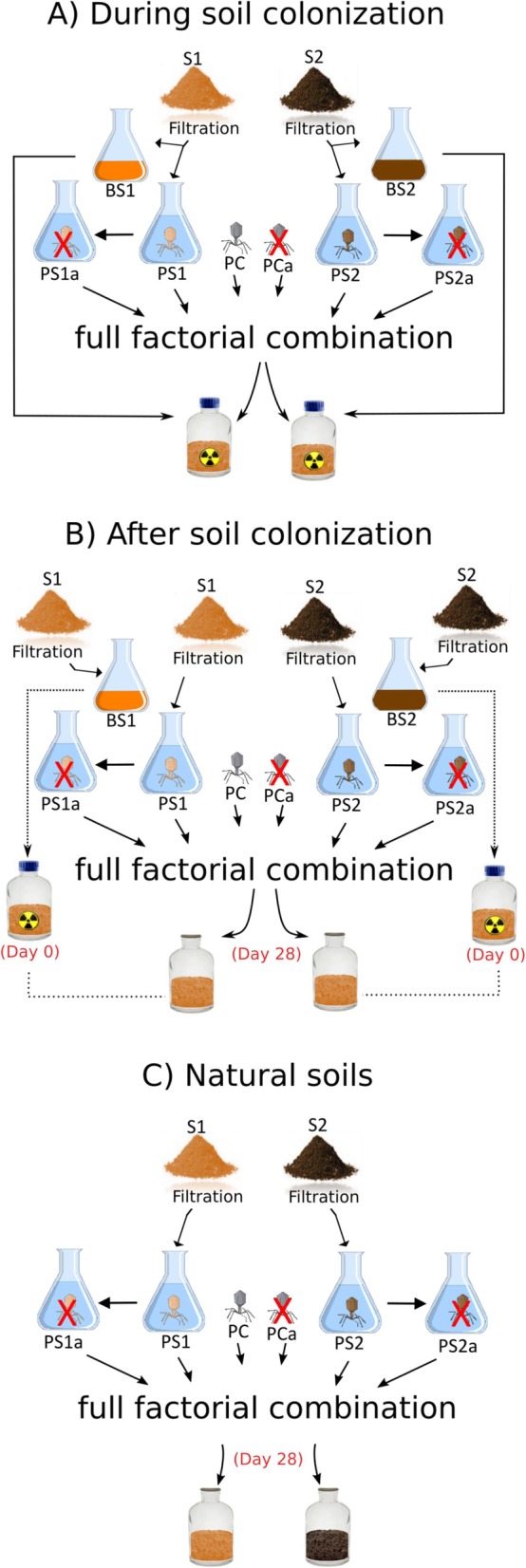
Schematic illustration of the experimental design. The microcosms were destructively sampled 34 days (**a** and **b**) or 35 days (**c**) after phage suspension inoculation. Soil suspensions from soil 1 (S1) and 2 (S2) were filtered to separate the phage fraction (PS1 and PS2) and bacterial fraction (BS1 and BS2). An outgroup with phage isolates named phage cocktail (PC) was also included. The different conditions were compared against a control made with autoclaved phage suspension (red cross) (PS1a, PS2a, and PCa). **a** During colonization experiment: both phage and bacterial suspensions were inoculated in microcosms made with sterile soil at the same time. **b** After colonization experiment: the bacterial fraction was inoculated first in the microcosms made with sterile soil, and the phage fraction was inoculated 28 days later. **c** Natural soils experiment: the phage fraction was inoculated in the microcosms made with the natural soils, 28 days after assembling the pots

The experimental conditions tested combined two soil bacterial communities (BS1 and BS2), three phage sources (PS1, PS2, and a phage cocktail named as PC, see “Materials and methods”), two phage suspension status (natural and autoclaved as control), and three community assembly experiments (A: *during colonization*, B: a*fter colonization*, and C: in *natural soils*) with five replicates. More details about the experimental design can be found in Fig. 1 and in the “Materials and methods” section.

**Fig. 2:**
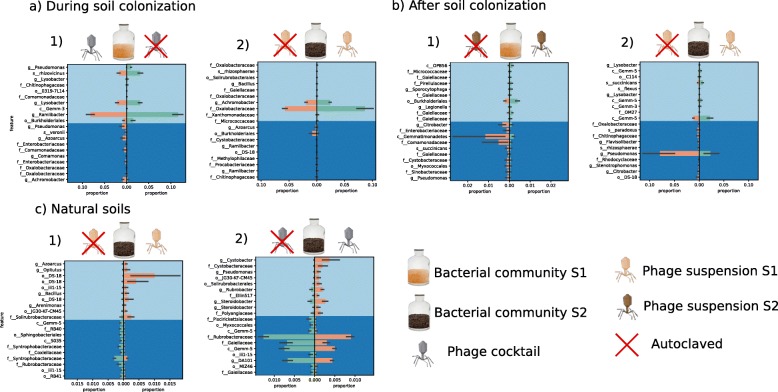
**a**–**c** Proportion plots of balance analysis from *gneiss* showing differentially abundant bacterial groups most affected by phage suspensions on treatments where both alpha and beta diversities where significantly changed (see Table 1). The plots are divided vertically comparing natural vs autoclaved (with red cross) conditions

Compared with the control conditions (i.e., autoclaved phage suspension), inoculation of phages during soil colonization resulted in significant differences in both alpha and beta diversities of the bacterial communities in a few treatments, i.e., BS1PC and BS2PS1 (Table 1). In BS1PC, phage inoculation increased the Shannon diversity (*p* value = 0.01), with significant differences in the Jaccard similarity index between the microcosms inoculated with the active and autoclaved phages (*p* value = 0.008). These changes were mainly associated with a reduction in the proportions of amplicon sequence variants (ASVs) assigned to *Ramlibacter*, *Lysobacter*, *Luteibacter*, *Burkholderiales*, and *Pseudomonas* (Fig. 2a.1). In contrast, phage inoculation in BS2PS1 decreased Shannon diversity (*p* value = 0.02) with significant differences in bacterial community composition based on Bray-Curtis dissimilarity (*p* value = 0.04). These changes were mainly related to an increase in the proportions of Oxalobacteraceae and *Achromobacter* and a decrease of Burkholderiales and *Azoarcus* (Fig. 2a.2).

**Table 1:** Significance of statistical tests for pairwise comparisons of community diversity metrics between natural vs autoclaved phage suspension

	Alpha diversity			Beta diversity			
	Faith-PD	Evenness	Shannon	UniFrac (unweighted)	UniFrac (weighted)	Jaccard (qualitative)	Bray-Curtis (quantitative)
During colonization							
BS1PS1	0.916	0.347	0.250	0.058	0.208	0.108	0.106
BS1PS2	0.754	0.250	0.174	0.705	0.462	0.442	0.185
BS2PS2	0.916	0.347	0.250	0.27	0.244	*0.017*	0.104
BS2PS1*	0.250	0.117	*0.028*	0.377	0.128	0.051	*0.045*
BS1PC*	0.464	*0.028*	*0.016*	0.058	0.083	*0.008*	*0.013*
BS2PC	0.462	0.806	0.624	*0.036*	0.123	*0.035*	0.104
After colonization							
BS1PS1	0.386	0.772	1	0.086	0.771	*0.027*	0.235
BS1PS2	*0.025*	0.654	0.101	0.057	0.217	0.554	0.263
BS2PS2	0.148	0.386	1	*0.027*	0.148	*0.025*	0.31
BS2PS1*	0.563	*0.043*	0.083	0.118	*0.024*	0.05	0.05
BS1PC	0.174	0.601	0.916	*0.041*	0.168	*0.035*	0.329
BS2PC	0.624	0.462	0.806	0.159	0.432	0.166	0.238
Natural soil							
S1PS1	*0.028*	0.754	0.075	0.112	0.178	0.136	0.229
S1PS2	0.327	0.327	0.624	0.756	0.181	0.76	0.734
S2PS2	0.456	0.296	0.654	0.711	0.671	0.752	0.739
S2PS1*	*0.025*	0.179	*0.025*	*0.017*	*0.049*	*0.026*	*0.049*
S1PC	0.086	0.05	0.05	0.852	0.695	0.098	0.268
S2PC*	0.086	*0.014*	0.141	0.072	*0.021*	*0.011*	*0.008*

Italicized *p* values < 0.05; asterisk (*) highlights treatments where both alpha and beta diversity were changed significantly (*p* values < 0.05) based on Kruskal-Wallis and PERMANOVA tests, respectively

The inoculation of phages after soil colonization by bacteria also showed a significant effect on both bacterial alpha and beta diversities when compared with the control conditions (i.e., autoclaved phage suspension) in the case of BS2PS1 (Table 1). Thus, inoculation of active phages resulted in significant differences in evenness (*p* value = 0.04) and weighted UniFrac (*p* value = 0.02) as well as in a decrease of *Pseudomonas* and Gemm-5 and an increase in *Flavobacterium* (Fig. 2b.2). In the other transplant microcosms, i.e., BS1PS2, only the decrease in Faith’s PD was significant (*p* value = 0.02). Similarly to the experiment performed during soil colonization, a significant effect of inoculation with the phage cocktail was observed on the BS1 community but only for two beta diversity metrics (unweighted UniFrac and Jaccard; Table 1).

In natural soils, active phages had the strongest impact on the bacterial communities in S2PS1 and S2PC (Table 1 and Fig. 2). In S2PS1, active phage inoculation led to significant differences in all beta diversity metrics with increased Faith’s PD (*p* value = 0.02) and decreased Shannon (*p* value = 0.02). These changes were mainly associated with an increase in proportions of ASVs assigned to DS-18 and with a decrease in proportions of Syntrophobacteraceae (Fig. 2c.1). In S2PC, differences in bacterial community composition based on Bray-Curtis (*p* value = 0.008) was concomitant to an increase in evenness (*p* value = 0.01) and a decrease in proportions of ASVs assigned to Rubrobacteraceae, Gemm-5, Gaiellaceae, and DA101 (Fig. 2c.2).

**Fig. 3:**
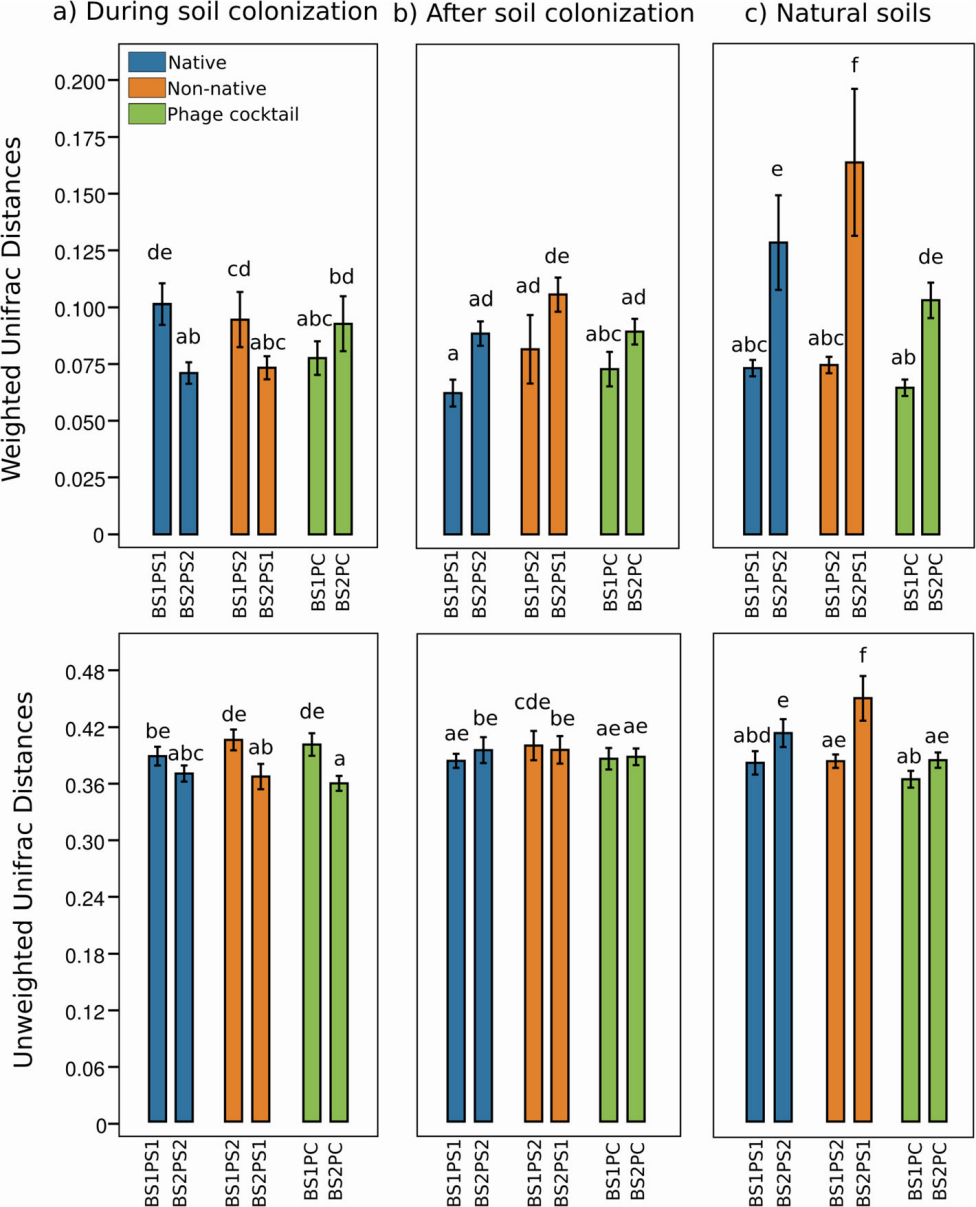
Weighted (**a**) and unweighted (**b**) UniFrac distance values between each treatment inoculated with active phages and the control inoculated with autoclaved phages. The values represent means with standard deviation and different letters above the bars indicate significant differences (Tukey’s test, *p* value < 0.05)

Overall, the major impacts of active phages were observed in bacterial communities from soil S2. In these conditions, the weighted UniFrac distance values increased significantly from experiments A to C when inoculated with non-native phages, being lower during colonization and higher in natural communities (*p* value < 0.05; Fig. 3). Furthermore, when comparing the conditions native vs non-native phages in natural soils, the weighted and unweighted UniFrac distance values were all significantly higher in S2 compared with S1 (*p* value < 0.05; Fig. 3ab). This is consistent with the significant differences observed in S2-derived communities inoculated with PS1 phages considering active vs autoclaved comparisons in experiments A, B, and C (Table 1; Fig. 2). The highest UniFrac distance values were obtained in S2PS1, being significantly different also when compared with S2PS2 (Fig. 3ab).

### Impact of phage cocktails on *Pseudomonas*, *Xanthomonas*, and *Bacillus*

As stated in the sections above, the microbiomes mostly impacted by PC (i.e., with significant differences in both alpha and beta diversity) compared to the control condition (i.e., autoclaved PC) were BS1 during soil colonization and the natural S2 community (Table 1 and Fig. 2). Because our phage collection was composed of phages isolated on *Pseudomonas*, *Bacillus*, and *Xanthomonas* strains, we checked whether ASVs belonging to those genera were impacted by PC. ASVs assigned to *Pseudomonas* were some of the most impacted phylotypes by addition of the active phage cocktails (Fig. 2a.1 and Fig. 2c.2). In experiment A BS1PC, we identified two *Pseudomonas* taxa which were significantly affected in presence of the phage cocktails, one negatively impacted while the other was positively impacted (Fig. 2a.1). In experiment C BS1PC, we found one *Pseudomonas* among the groups positively impacted by phage cocktails (Fig. 2c.2). *Xanthomonas* and *Bacillus* were not detected as part of the bacterial groups most impacted by PC.

### Network models

**Fig. 4:**
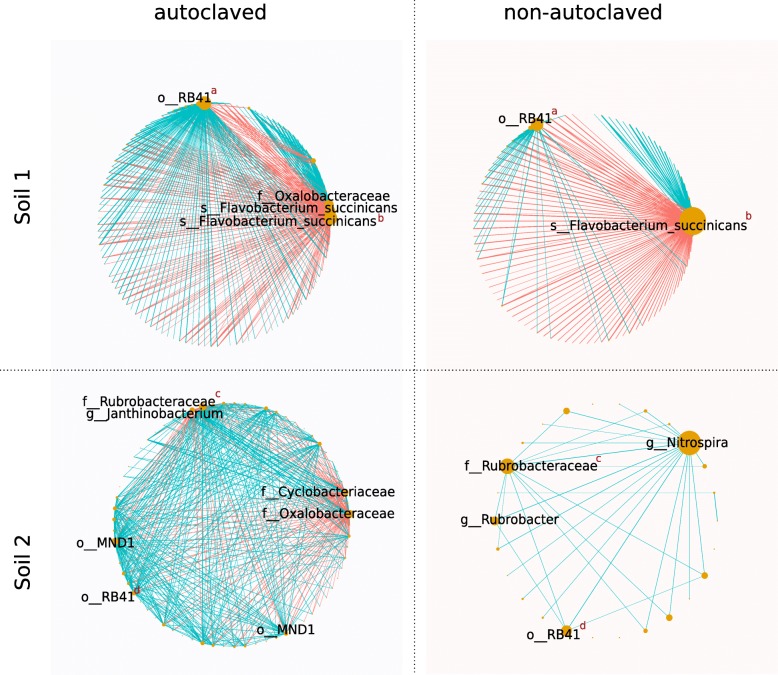
Poisson lognormal model for sparse covariance inference for bacterial abundances. Nodes represent ASVs sized based on degree. Equal superscript red letters indicate that nodes in different networks correspond to the same clade (i.e*.*, same ASV). Edges connecting nodes represent positive (light blue) and negative (red) interactions

**Fig. 5:**
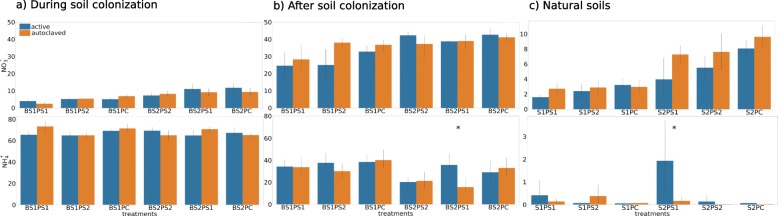
Soil inorganic nitrogen (N) determination. *Y*-axis indicates NO_3_^-^ and NH_4_^+^ by mg N/kg of dried soil. *X*-axis indicates treatments. BS1: bacterial community from soil 1; BS2: bacterial community from soil 2; S1: natural soil 1; S2: natural soil 2; PS1: phage community from soil 1; PS2: phage community from soil 2; PC: phage cocktail. *Y*-axis scale of the natural soils (**c**) is different from the other plots (**a** and **b**). *Significant differences in pairwise comparisons (*n* = 5) between natural phage suspension (blue) vs control autoclaved phage suspension (orange) (Tukey’s HSD test, *p* ≤ 0.05)

Our network models provide evidence that phages impacted associations between bacterial groups (Fig. 5). Models representing microbial systems inoculated with phage suspensions showed lower degree of complexity compared to models inoculated with autoclaved suspensions for both S1 and S2 (Additional file 3: Table S1). Comparison between the autoclaved vs non-autoclaved phage network models revealed that the overall number of connections decreased for both S1 and S2 in presence of active phages (Fig. 4). In S1, the degree of a node representing a *Flavobacteruim* in the network models increased with active phage inocula compared to the control condition while the number of hubs (i.e., nodes with higher degree of connections in the models) decreased (Fig. 4). In S2, a similar effect was observed with *Nitrospira*, *Rubrobacter*, and a Rubrobacteraceae species (Fig. 4).

### Soil inorganic nitrogen

Quantification of inorganic nitrogen pools showed large differences between natural soils and sterile soils (Fig. 5). Natural soils presented a distinct pattern with much lower concentrations of NO_3_^-^ and NH_4_^+^ compared to experiments in sterile soils (*during colonization* and a*fter colonization* experiments). During soil colonization, NO_3_^-^ concentrations ranged between 2.49 and 11.72 mg of N per kilogram of dried soil while the NH_4_^+^ ranged between 65 and 73 mg of N per kg of dried soil, without any significant effect of phage inoculation. After soil colonization, NO_3_^-^ concentrations ranged between 24 and 42 mg of N per kilogram of dried soil with a NH_4_^+^ concentration in the same range. A significant effect of phage inoculation on the inorganic nitrogen concentration was observed in the experiment testing phage inoculation after soil colonization for the BS2PS1 treatment. Thus, inoculation with PS1 resulted in more than a two-fold increase in the NH_4_^+^ concentration (Tukey’s HSD test, *p* values = 0.05, Fig. 5). Similarly, the NH_4_^+^ concentration increased significantly in natural soil 2 also when inoculated with PS1 when compared to the autoclaved phage control, resulting on average in a ten-fold increase (Tukey’s HSD test, *p* values = 0.02; Fig. 5).

## Discussion

Compared to other ecosystems, the role of phages on soil microbial communities has been overlooked [25, 31] and soil phages are now being studied mostly through metagenomic approaches [22, 23]. In the present study, we performed several manipulation experiments aiming to achieve a better understanding of the importance of phages for soil bacterial community assembly and functions. Although it does not fully reflect the complexity of soil communities or of their natural habitat, our microcosm experiments have provided some insights into the ecological role of phages in soil. The viral fractions were obtained by filtering the soil suspensions and subsequently used as inoculants in the microcosm experiments (Fig. 1). The viral fraction selected for this experiment was based on a size range of 100 kDa to 0.2 μm. Given that cell size distributions of soil bacteria is in general much larger than 0.22 μm, less than 3% of bacterial cells are expected to fall below this threshold [32]. On the other hand, some phage particles can exceed 0.2 μm [33], although the majority of phages examined by electron microscopy so far belong to the caudovirales order, with capsid sizes < 0.22 μm [34]. However, whether Caudovirales are predominant in soil is unclear [35–38]. Our TEM analysis confirms the integrity of phages in both PS1 and PS2 filtered suspensions, as well as the presence of *Caudovirales* phages (Additional file 2: Fig. S2ab). However, the majority of phage populations represented by the bins recovered from the soil-filtered suspensions could not be assigned to known phage clades, which indicates that the phages present in the soil suspensions have not been described yet. This was expected since viruses are considered as the main contributors to the biological dark matter due to the considerable proportion of viral sequences in metagenomic datasets without any similarity to sequences in public databases [23, 39–41]. Our analysis revealed that the diversity of both phages and bacteria was higher in soil S1 than S2 (Additional file 1: Fig. S1 and Additional file 2: Fig. S2c; *p* values < 0.05), which is in agreement with previous studies suggesting that parasite diversity is strongly correlated with host diversity [42, 43]. The low abundance of bacterial cells in the phage suspensions observed by TEM was confirmed by metagenomic analysis. Thus, more than 97.8 % of mapped reads were phage genomic sequences. Based on the taxonomy of bacterial bins, none of them were related to taxa that were significantly affected by phage inoculation compared with autoclaved controls, which suggests that the few small bacterial cells remaining in the phage suspensions have not hampered our approach. Nevertheless, it should be noted that an unknown proportion of lysogens and filtrable phages adsorbed to host cells may potentially contribute as a source of phages in the bacterial suspensions after filtration, which could lead to an underestimation of the phage importance in our experiment. Such partitioning effect might not be the same in different soil communities, and this factor should also be considered for explaining why the treatment effect in S1 was observed in a lesser extent compared to S2 (Table 1).

Phage-bacteria arms race is established in the context of local adaptation [44, 45]. Thus, bacterial hosts can evolve various mechanisms to resist the attack of phages present in their environment [46]. For instance, Buckling and Rainey [47] incubated coevolving *Pseudomonas* and its phage derived from the same populations, but separated in different microcosms, and verified that the bacterial population was better able to resist phages coevolving locally than phages that coevolved with the *Pseudomonas* in a separated microcosm. We therefore hypothesized that bacterial communities would be more affected by their non-native phages than phages obtained from the same soil. Accordingly, the largest effects of active phages were observed when inoculating PS1 into the natural soil S2 (experiment C) and to a lesser extent, although still significant, into BS2 during and after soil colonization by bacteria (experiments A and B), with shifts in both alpha and beta diversities of bacterial communities (Table 1). The inoculation of native phages resulted in weaker effects, which were significant only for some alpha or beta diversity indices (Table 1; *p* value = 0.05). On the other hand, no clear effects of native vs non-native phages were observed for PS2 (Table 1), indicating that the local adaptation context cannot fully explain our data. This observation supports the hypothesis that diversity of both phages and hosts (Additional file 1: Fig. S1 and Additional file 2: Fig. S2c; *p* values < 0.05) can be of importance for determining the phage impact in soil microbial communities due to differences in bacteriophage host range and bacterial resistance between the soils. While we hypothesized that already-established bacterial communities can explore the soil spatial structures and find shelter in biofilms or other aggregates, we did not find consistent differences when phages were inoculated in soil during (experiment A) and after colonization (experiment B) (Table 1). That significant effects of phage inoculation were observed in natural soil also contradicts our second hypothesis that already-established communities would be more protected from phage predation (*p* values < 0.05; Table 1). Nevertheless, the significant differences observed across the experiments depending on the inoculation time (Table 1) suggest that phage inoculation time matters for the soil microbial community assembly process. This is for example of interest for the potential use of phage as plant disease biocontrol agents [13].

Our results also demonstrate that a cocktail of a few phages was sufficient to affect a complex bacterial community not only when inoculated in a sterile soil together with the bacteria (BS1PC) but also when inoculated into a natural soil (S2PC) (Table 1), with a stronger effect in S2PC (Fig. 3). The phage cocktail was made of five phages, three of them having *Pseudomonas* as hosts, the others *Xanthomonas* and *Bacillus*. Inoculation with the phage cocktail did significantly decrease the relative abundance of one *Pseudomonas* strain but surprisingly also increased the relative abundance of two other *Pseudomonas* strains (Fig. 2a.1). A hypothesis to explain this results is that the two *Pseudomonas* strains that increased in abundance are resistant to the phages but outcompeted by the *Pseudomonas* strain susceptible to phages through intraspecific competition [48, 49]. As such, the killing of the susceptible *Pseudomonas* strain resulted in an increased fitness of the other two *Pseudomonas* strains. On the other hand, the lack of significant changes in *Bacillus* and *Xanthomonas* can be explained by narrow host range of the *Bacillus* and the *Xanthomonas* phages [11, 50, 51] or outright immunity to these phages. In contrast, in the natural soil (S2PC), no *Pseudomonas*, *Xanthomonas*, or *Bacillus* ASVs were detected among the taxa most impacted by the phage cocktail (Fig. 2c.2), which might be explained by differences in host availability or soil composition.

At the bacterial community level, phages are expected to have both direct and indirect effects [1, 2]. Direct effects arise as a consequence of lytic infections that control the abundance of host populations [1–3], while indirect effects arise as a consequence of changes in the balance of interspecific interactions between bacteria due to the killing by phage of susceptible competitors/mutualists and by the biomass turnover (i.e., viral shunt) [2, 48, 52]. The association network models built in this study (Fig. 4) showed that the presence of active phages decreased the connections between nodes as well as the number of hub nodes (Additional file 3: Table S1; Fig. 4). If we assume that habitat variability was reduced in our microcosm experiment without differences in abiotic factors between microcosms inoculated with active and autoclaved phages, the effects of niche preference that can be a source of co-occurrence patterns in inferred networks were limited in our experiment. Therefore, the models constructed in this study (Fig. 5) provide evidence that phage pressure changes the balance of interactions between bacteria with a major effect of phages in soil S2.

Our results also provide some evidence that phage addition may not only affect soil bacterial communities but also microbe-mediated processes such as N-cycling (Fig. 5). Addition of phages had no impact on inorganic N pools in most treatments except in natural soil S2 and in the already colonized soil BS2 when inoculated by the phage suspension from S1 (Fig. 5). Interestingly, these treatments were among those exhibiting the strongest impact of phages on bacterial diversity (Table 1 and Fig. 3). These results add evidence to a growing body of literature reporting a relationship between microbial diversity and ecosystem functioning [53]. For example, Calderón et al. [54] observed that changes in soil microbial diversity significantly impacted NH_4_^+^ concentrations. Alternatively, the increased NH_4_^+^ concentration could also be due to the lysis of host cells by phages, resulting in a release of inorganic nitrogen followed by mineralization. This is supported by the findings of virus-mediated release of organic nitrogen during cell lysis in the ocean. Shelford et al. [55] determined the efficiency of lysate remineralization and transfer to phytoplankton, reporting an uptake of ^15^N of 0.09 to 0.70 μmol N μg^−1^ of chlorophyll after addition of ^15^NH^+^_4_ labeled lysate from a *Vibrio* sp. That phage addition in some treatments resulted in significant shifts in bacterial community composition but not in inorganic nitrogen content could be explained by the fact that the cells killed were not important players in N-cycling. In any case, our findings suggest that changes in soil bacterial community due to an increase in phage pressure can affect microbial-driven functions.

## Conclusion

Overall, the results presented here demonstrate that increase in phage pressure can impact the assembly of soil bacterial communities, as well as their activities. However, for the different community assembly scenarios, we found important discrepancies depending on the microbiota, which suggests that host community diversity and composition are important factors determining the phage impact. Our results emphasize the importance to take into account the effect of phages on soil bacterial communities for better understanding the dynamic of these communities.

## Material and methods

### Soil sampling and chemical properties

Two different agricultural soils were selected for our experiment. Soil 1 (S1) was sampled at INRA’s experimental station in Dijon (47° 30′ 22.1832″ N, 4° 10′ 26.4648″ E), France. While soil 2 (S2) was sampled at INRA’s experimental station in Montpellier (43° 37′ 04.7″ N, 3° 51′ 26.2″ E), France. S1 soil properties were 41.9% clay, 51.9% silt, 6.2% sand, 2.6 % of organic matter (OM), 5.6 pH, and 18.9 cmolc kg^−1^ cation exchange capacity (CEC). S2 soil properties were 28.8% clay, 35.2% silt, and 34.6% sand, 1.2% OM, pH of 8.6, and 11 cmolc kg^−1^CEC. Samples were collected with an alcohol sterilized soil auger. Each soil was sieved (4 mm, alcohol sterilized) and stored at − 20 °C before downstream procedures.

### Experimental design

To investigate the effect of soil phage inoculation on soil bacterial community composition and diversity as well as inorganic N pools, we performed experiments using a reciprocal transplant design under different community assembly scenarios. The different community assembly scenarios assessed whether colonizing, established, or natural bacterial communities are affected in a similar way by native or non-native phages. We incubated soil phage suspensions derived from soils S1 and S2 (namely PS1 and PS2) with their native or non-native communities in sterile soils or natural soils. Soil phage suspensions were obtained using a tangential filtration systems which will be explained in details further in this section. The retentates (i.e., bacterial suspensions, namely BS1 and BS2) were used for experiments in sterile soil. An additional treatment based on a mixture of previously isolated phages (namely phage cocktail; PC) was included as an outgroup. The resulting experimental conditions were:

- *Phage and bacteria suspensions inoculated in sterile soil at the same time*. Microcosms containing sterile soil were inoculated with BS1 or BS2 suspension and either (i) the phage suspensions from the same soil, (ii) the phage suspension from the other soil, or (iii) the cocktail of phage isolates.
- *Phage suspensions inoculated after bacteria inoculation in sterile soil*. Microcosms containing sterile soil were inoculated with BS1 or BS2, and after 28 days, they were inoculated with either (i) the phage suspensions from the same soil, (ii) the phage suspension from the other soil, or (iii) the cocktail of phage isolates.
- *Phage suspensions inoculated in natural soils*. Microcosms containing non-sterile soil (S1 or S2) were setup, and after 28 days of incubation, they were inoculated either by (i) the phage suspension from the same soil, (ii) the phage suspension from the other soil, or (iii) the cocktail of various phage isolates.

Overall, the experimental conditions tested included two soil bacterial communities (BS1 and BS2) × three phage sources (PS1, PS2, and PC) × two phage suspension status (natural and autoclaved as control) × three community assembly experiments (A: *during colonization*, B: *after colonization*, and C: in *natural soils*) × five replicates (i.e., *n* = 5), giving a total of 180 microcosms (Fig. 1). The IDs for each treatment hereinafter will be named as following for each community assembly experiment (A, B, or C): source of hosts (BS1 or BS2 for bacterial suspensions, and S1 or S2 for natural soils): phage origin (PS1, PS2, or PC) and phage status (ending by “a” if autoclaved), e.g., BS1PS2 or BS1PS2a.

### Preparation of the phage and bacterial suspensions from soil S1 and S2

The TFF system is useful to filter large amounts of environmental samples more efficiently compared to conventional perpendicular filtration systems, in which soil particles block membrane pores more easily. We used two filters (MiniKros® Sampler polysulfone provided by Repligen) with different pore sizes: 0.2 μm and 100 kDa. They were assembled in parallel using a peristaltic pump (MiniPlus 3, Gilson) running at constant flux (10 rpm) adjusted to filter at minimum pressure. Filters were sanitized according to the manufacturer instructions using 0.1 M NaOH solution previously to each sample filtration.

Soil suspensions derived from 4 kg of soil (S1 or S2) washed with 4 L of phage buffer (68 mM NaCl, 10 mM MgSO_4_, Tris-Cl pH 7.5) [56], by dividing it into 500-mL bottles, that were manually shaken before centrifuging at 4500 G for 20 min at 4 °C, and then filtered with a TFF system. The retentates obtained (BS1 or BS2) were used in experiments A and B, and the filtrates (PS1 or PS2) were used in experiment A. After the incubation period (Fig. 1), this procedure was repeated and the filtrates obtained (PS1 or PS2) were used in experiments B and C. The final volumes of filtrates and retentates were 500 mL and 1.5 L, respectively. They were stored in 4 °C and inoculated in the different microcosms on the following day.

### Preparation of the phage cocktail suspension

The phage cocktail (PC) was obtained by mixing three *Pseudomonas* phages, two of them isolated using *Pseudomonas syringae* pv. tomato as host (~ 10^7^ plaque-forming units (PFUs) both) and one using *Pseudomonas syringae* pv. avii (~ 10^8^ PFUs), one *Xanthomonas* phage (~10^9^ PFUs), using *Xanthomonas campestri* pv. citri as host, and one *Bacillus* phage (~10^6^ PFUs), using a *Bacillus simplex* as host. The latter was isolated from S1 by L.P.P. Braga. The other phages were isolated from plant decomposing material by W. Kot and L.H. Hansen in Denmark. Phage isolates were isolated by enrichment method using double-layer agar followed by plaque purification [57]. The PC inoculant was made of 400 μL of lysates of each phage, a total volume of 2 mL (i.e., five phages × 400 μL).

### Microcosms setup

The experiments A (during colonization) and B (after colonization) were performed using microcosms containing 50 g of dry soil S1 sterilized by gamma-radiation (35 kGy; Conservatome, Dagneux, France). The experiment C (natural soils) was performed using microcosms containing 50 g of soil S1 or S2. The volumes inoculated in the microcosms were 6 mL of PS1 or PS2 and 18 mL of BS1 or BS2. They were sampled from the final volumes obtained with TFF (retentate or filtrate). The bottle was vigorously shaken prior to each sampling. Autoclaved phage suspension PS1, PS2, or PC were included as controls. All microcosms were incubated at room temperature in sterile conditions for 34 to 35 days after phage inoculation, and moisture was maintained at 80 % of field capacity by regular addition of sterile water.

### Transmission electron microscopy

In order to qualitative confirmation of the presence and integrity of phage particles in soil-filtered suspensions, microscopy images were obtained by TEM performed at the Center of Microscopy, INRA, Agroecology (Dijon). Five microliters sampled direct from phage suspensions (PS1 or PS2) were adhered to cooper EM grid overlaid with a collodion carbon film for 1 min, excess solution wicked of with a filter paper. The grid was stained with 2% uranyl acetate for 1 min and airdried. Grids were observed with a Hitachi H7500 (Hitachi Scientific Instruments Co., Tokyo, Japan) transmission electron microscope operating at 80 kV and equipped with an AMT camera.

### DNA extraction and sequencing

DNA was extracted from samples of PS1 and PS2 that were collected in duplicates. Briefly, 1 mL of a given phage suspension was sampled and 15 U of DNase I was added to 900 μl of filtrate before incubating for 30 min at 37 °C to remove residual DNA. Then SDS (final concentration of 0.2%) and 30 μl of proteinase K (20 mg/ml) were added, followed by incubation for 1 h at 55 °C. Phage DNA was purified using Clean & Concentrator-10 kit (Zymo Research, CA, USA) according to the manufacturers’ protocol. The sequencing libraries were built using Nextera XT kit (Illumina, CA, USA) and sequenced on a NextSeq platform using 300 cycle MID kit v.2 which gives paired-ended reads of 150 bp. Each sample was sequenced four times to enable phage genome recovery from the dataset.

Soil DNA extraction was performed using DNeasy PowerSoil HTP 96 Kit (Qiagen, Hilden, Germany) with 0.3 g of soil from each soil microcosm. Samples from the initial non-treated soils (S1 and S2) were also included (*n* = 5). DNA quantification after extraction was performed with picogreen. 16S rRNA gene amplicons were generated in two steps according to Berry et al. [58]. In the first step, the bacterial 16S rRNA gene V3-V4 hypervariable region was amplified by polymerase chain reaction (PCR) using the fusion primers U341F (5′-CCTACGGGRSGCAGCAG-3′) and 805R (5′-GACTACCAGGGTATCTAAT-3′) (Takahashi et al. 2014), with overhang adapters (forward: TCGTCGGCAGCGTCAGATGTGTATAAGAGACAG, adapter: GTCTCGTGGGCTCGGAGATGTGTATAAGAGACAG) to allow the subsequent addition of multiplexing index-sequences. PCR was carried out in duplicate 15 μL reactions containing 7.5 μL Phusion High-Fidelity PCR Master Mix (Thermo Fisher Scientific, MA, USA), 0.25 μM of each primer, 250 ng T4 gp32 (MPBio), and 1 ng template DNA. Thermal cycling conditions were 98 °C for 3 min followed by 25 cycles of 98 °C for 30s, 55 °C for 30s, and 72 °C for 30s, with a final extension at 72 °C for 10 min. Duplicated first step PCR products were pooled then used as template for the second step PCR. In the second step, PCR amplification added multiplexing index-sequences to the overhang adapters using a unique multiplex primer pair combination for each sample. The reaction was carried out in duplicate 30 μL volumes containing 15 μL Phusion High-Fidelity PCR Master Mix (Thermo Fisher Scientific, MA, USA), 1 μM of one forward and one reverse multiplex primer, and 6 μL of first step PCR product. Thermal cycling conditions were 98 °C for 3 min followed by 8 cycles of 98 °C for 30s, 55 °C for 30s, and 72 °C for 30s, with a final extension at 72 °C for 10 min. Duplicate second step PCR products were pooled then visualized in 2% agarose gel to verify amplification and size of amplicons (around 470 bp). The amplicons were cleaned-up and pooled using sequalPrep^TM^ Normalization plate kit 96-well (Invitrogen). Sequencing was performed on MiSeq (Illumina, CA, USA; 2 × 250 bp) using the MiSeq reagent kit v2 (500 cycles). Demultiplexing and trimming of Illumina adaptors and barcodes was done with Illumina MiSeq Reporter software (version 2.5.1.3).

### Soil inorganic nitrogen determination

Mineral nitrogen pools (NO_3_^-^ and NH_4_^+^) present in the soil were quantified according to the ISO standard 14256-2. Quantification was performed using three blanks in each series by colorimetry in a BPC global 240 photometer. Statistical test for detecting differences in levels of NH_4_^+^ and NO_3_^-^ across soil samples was performed in R environment using ANOVAs (*aov* function) followed by Tukey’s honestly significant difference (HSD) test, both from the *stats* package.

### Computational and statistical analyses

Metagenomic sequences of phage suspensions from S1 and S2 were assembled with MetaSPADES separately [59]. Assembled sequences were mapped using BWA [60] and SAMTOOLS [61] was used to process the mapped data. Binning was performed with MetaBAT2 [62], MaxBIN [63], and CONCOCT [64] using MetaWRAP [65]. The bins found were dereplicated with DREP [66]. Phage bins were identified using a machine learning method implemented by MARVEL [67], because it uses bins to make predictions and demonstrated higher recall rates compared with other available tools. Next, relative abundance of phage bins was calculated with MetaWRAP [65] by the function *quant_bins* using Salmon [68] that quantifies the metagenomic reads directly against the bins. The mapping procedure is based on an auxiliary k-mer hash and was performed according to the default parameters, considering k-mers of length 31 as the minimum acceptable length for a valid match. The MetaWRAP function calculates the relative abundance normalized according to the size of the sequences and the portion of mapped reads in the dataset [65, 68]. Statistical test for detecting differences in phage bin abundances across soil samples was performed using the python function *scipy.stats.ttest_ind*. Taxonomic classification of phage bins was investigated with vConTACT2 using the ProkaryoticViralRefSeq88 database [69]. Open reading frames (ORFs) in phage bins were identified with MetaProdigal [70]. Contigs containing bacterial genomic sequence were considered, and bins were analyzed with CheckM [71] for further checking possible contamination.

16S rRNA gene amplicon analysis was performed in QIIME2 [72] environment. Sequences were filtered with Trimmomatics [73] and Cutadapt [74] for removal of illumina artificial sequences and low-quality sequences. The data set was imported into QIIME2, and paired-end reads were joined with VSEARCH [75] following *q-score*-*joined* method. Construction of the feature table based on amplicon sequence variants (ASVs) was performed using Deblur pipeline [76], which removes reads presenting more than two probable erroneous base calls, denoises, dereplicates, and filter chimeras. The average number of reads per sample after denoising was 6018 (± 2821). Rarefaction thresholds were determined for each pairwise within-group comparison separately, i.e., autoclaved vs active. For an optimal rarefaction threshold, one or two replicates had to be discarded in the following treatments: BS2PC (*n* = 4), from *During Colonization Experiment*; BS1PS2 (*n* = 3), BS2PS2 (*n* = 4), BS2PS2a (*n* = 4), BS2PS1 (*n* = 4), BS2PS1a (*n* = 4), BS2PCa (*n* = 4), from *After Colonization Experiment*; and BS1PS2 (*n* = 4), S2PS2 (*n* = 3), S2PS1 (*n* = 3), BS1PC (*n* = 4), and BS2PC (*n* = 4), from the *Natural Soils Experiment*. Tree for phylogenetic diversity analysis was built using the methods implemented in the *align-to-tree-mafft-fasttree* pipeline, with MAFFT [77] and FastTree [78]. Taxonomy was assigned by a classifier trained on V3–V4 region, including the set of primers used, based on greengenes database (v.05/2013 [79]). Next, diversity analyzes were performed according to the methods implemented by the *core-metrics-phylogenetic* pipeline following PERMANOVA and Kruskal-Wallis pairwise tests for assessing statistical significance on beta and alpha diversity tests, respectively. Significant differences in community diversity (*p* value ≤0.05) were further investigated with the *gneiss* pipeline to assess relevant microbial taxon contributing to the changes. Weighted and unweighted UniFrac distance values from the matrices that were obtained for within-group comparison were extracted in QIIME1 [80] to enable a between-group comparison across the experiments. The bar plots representing these values were obtained in PAST3 [81], and the statistical tests were performed using *multcomp* Tukey’s test in R environment.

Network models were constructed to investigate possible phage-derived indirect effect on microbial groups that could be expected due to elimination of competitors, nutrient release, generalized transduction, or AMGs inputs. The models were constructed with r package PLNmodels [82]; sparse inverse covariance estimation was calculated using default parameters; the best model was extracted with the function *getBestModel* and analyzed in *igraph* [83] using ASVs count tables pooling S1 and S2 samples separately to compare autoclaved vs non-autoclaved conditions.

## Supplementary information

**Additional file 1.** Fig. S1.

**Additional file 2.** Fig. S2.

**Additional file 3.** Table S1.

## Data Availability

Datasets are publicly available at the Sequence Read Archive—NCBI (PRJNA550482 and PRJNA550474).
