# Supplementary figures and images for: Impact of phages on soil bacterial communities and nitrogen availability under different assembly scenarios
Source: Microbiome. 2020 Apr 6;8:52. doi: 10.1186/s40168-020-00822-z (PMC7137350; doi:10.1186/s40168-020-00822-z)

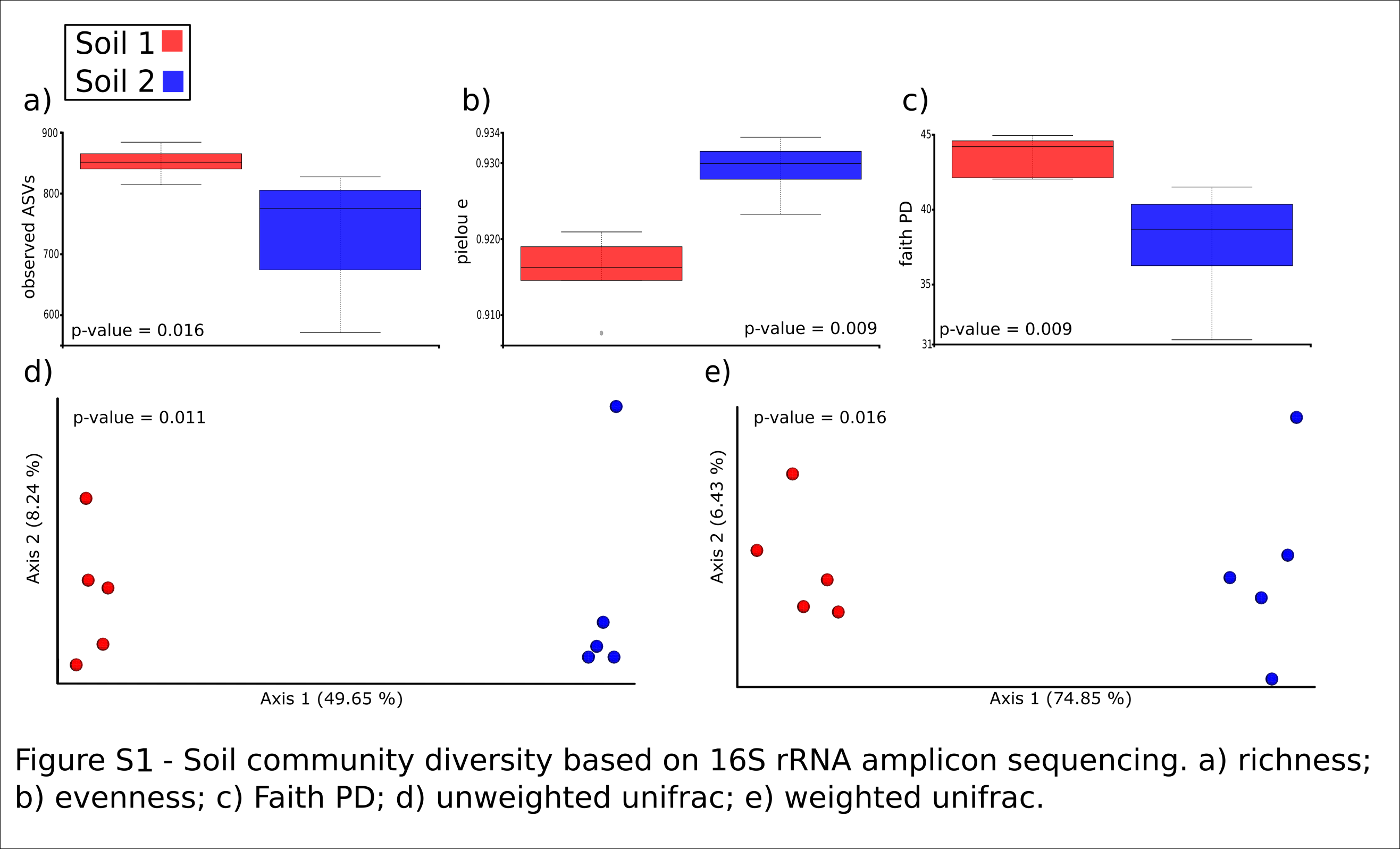

Supplement: Supplementary file 2 — Additional file 1. Fig. S1. [file 40168_2020_822_MOESM1_ESM.png]
