# Supplementary material for: Impact of phages on soil bacterial communities and nitrogen availability under different assembly scenarios
Source: Microbiome. 2020 Apr 6;8:52. doi: 10.1186/s40168-020-00822-z (PMC7137350; doi:10.1186/s40168-020-00822-z)
Supplement: Supplementary file 4 — Additional file 3. Table S1. [file 40168_2020_822_MOESM3_ESM.docx]

Table S1 – Network topology

| graph | number of nodes | number of edges | density | modularity | number of clusters* |
| --- | --- | --- | --- | --- | --- |
| S1 active phage suspension | 94 | 192 | 0.005 | 0.26 | 3 |
| S1 phage suspension autoclaved | 114 | 434 | 0.007 | 0.17 | 4 |
| - | - | - | - | - | - |
| S2 active phage suspension | 26 | 53 | 0.001 | 0.2 | 4 |
| S2 phage suspension autoclaved | 69 | 484 | 0.008 | 0.2 | 2 |

*Louvain algorithm
